# MAPT Haplotype Variation and Alzheimer’s Disease Risk: A Narrative Review with Focus on the Jordanian Population

**DOI:** 10.2174/011570159X371971250818110608

**Published:** 2025-09-10

**Authors:** Alaa A. A. Aljabali, Almuthanna K. Alkaraki, Mohammad A. Obeid

**Affiliations:** 1 Department of Pharmaceutics and Pharmaceutical Technology, Faculty of Pharmacy, Yarmouk University, P.O. Box 566, Irbid, 21163, Jordan;; 2 Department of Biological Sciences, Faculty of Science, Yarmouk University, Irbid, 21163, Jordan

**Keywords:** MAPT haplotypes, Alzheimer’s disease, tau protein, H1 and H2 haplotypes, Jordan, population genetics, genetic risk modeling, neurodegeneration

## Abstract

**Introduction:**

Genetic variations in the microtubule-associated protein tau (MAPT) gene play a central role in Alzheimer's disease (AD) pathogenesis. Two major MAPT haplotypes, H1 and H2, show differential associations with tau expression and AD risk. However, data from Middle Eastern populations remain limited, restricting our understanding of population-specific disease susceptibility patterns and therapeutic responses.

**Methods:**

We conducted a comprehensive literature review using PubMed, Scopus, and Web of Science databases. Search terms included “MAPT haplotype,” “Alzheimer's disease,” “H1 H2,” “tau pathology,” and “pharmacogenetics.” We analyzed peer-reviewed articles published between 2000 and 2024, focusing on studies reporting haplotype frequencies, MAPT expression levels, APOE interactions, and clinical outcomes. This review synthesizes published data without generating new experimental results.

**Results:**

The H1 haplotype consistently associates with increased MAPT expression, tau accumulation, and elevated AD risk, particularly in APOE ε4 noncarriers. Conversely, the H2 haplotype appears protective, correlating with reduced tau burden and slower cognitive decline. Notably, recent reports reveal significant overrepresentation of the H2 haplotype in the Jordanian population compared to European and East Asian cohorts, where H2 frequency is substantially lower or absent. This distinct genetic architecture suggests altered regional AD risk profiles.

**Discussions:**

The elevated H2 frequency in Jordan represents a unique population-specific genetic signature that may influence regional AD susceptibility patterns. These findings challenge current risk models predominantly based on European populations and suggest the need for population-tailored approaches in neurodegenerative disease research. The naturally H2-enriched Jordanian cohort provides an exceptional opportunity to investigate protective mechanisms against tau pathology.

**Conclusion:**

MAPT haplotype distributions show significant population variation with important implications for AD risk assessment and therapeutic targeting. The high H2 frequency in Jordan warrants integration into personalized medicine frameworks and population-specific disease models, potentially informing more effective regional prevention and treatment strategies.

## 1 INTRODUCTION TO MAPT AND ITS GENETIC INFLUENCE ON ALZHEIMER'S DISEASE

This review compiles and contextualizes published genetic evidence on MAPT haplotypes, particularly regarding AD risk and their proposed relevance to therapeutic implications [1]. Emphasis is placed on population-specific genetic distributions, drawing from existing literature, including emerging data from Middle Eastern cohorts. The gene encoding microtubule-associated protein tau (MAPT) is located on chromosome 17q21.31 and plays a critical role in neurodegenerative conditions, such as Alzheimer's and Parkinson's disease [2]. At this locus, two major haplotypes, H1 and H2, are defined as variants within a large inversion on the chromosome in the case of MAPT [3]. This inversion is associated with differential gene expression that links the H1 haplotype with increased MAPT expression and increased risk of tau pathology, whereas the protective function of the H2 haplotype retains the expression of MAPT at a moderate level. Elucidation of these haplotypes and their role in modifying AD risk is relevant to genetic research and therapeutic advances in neurodegenerative diseases [4].

In addition to genetic factors, neurodegenerative diseases also reflect accumulated cellular stress and impaired repair mechanisms. Mitochondrial decline, oxidative imbalance, reduced autophagy, and low-grade chronic inflammation, known as inflammaging, are linked to disease progression [5, 6]. Nutrient status and redox-active natural molecules also play important roles. These compounds can activate the hormetic stress pathways. This helps to alter antioxidant defenses and reduce inflammation in certain cell areas. Studying these physiological processes with MAPT haplotype variation will help us to understand how genes and metabolism interact. These interactions can influence the disease risk and progression [7, 8]. This review is narrative in design and does not involve original data generation or statistical analysis.

To date, no comprehensive haplotype analysis of MAPT has been conducted in a Jordanian population. Our results reveal a distinctive genetic architecture, characterized by H2 overrepresentation, that may have functional consequences for AD risk and therapeutic planning in this regional context [9]. To date, no focused analysis has examined the MAPT haplotype distribution in the Jordanian population, despite known geographic variability and its potential relevance for region-specific genetic susceptibility models.

Despite considerable research on the genetic architecture of Alzheimer’s disease, data from Middle Eastern populations remain severely underrepresented. A recent global meta-analysis of MAPT haplotype studies reported that Middle Eastern cohorts comprised less than 0.5% of all participants included in genotype-phenotype investigations of the H1 and H2 lineages [4]. This striking lack of representation has created substantial gaps in our understanding of haplotype-related risk distribution and has limited the development of inclusive genetic risk models. In parallel, large-scale efforts to map population-specific structural variants have identified significant regional diversity in the MAPT locus architecture, including potential sub-haplotype shifts that remain unexplored in Arab populations [10]. Therefore, there is a pressing need to document and contextualize MAPT haplotype frequencies within Middle Eastern groups to address global disparities in genetic epidemiology and inform emerging strategies in precision medicine. Recent global meta-analyses have consistently underrepresented Middle Eastern cohorts in MAPT haplotype studies, accounting for less than 0.5% of the total genotype data [4]. The present review addresses this gap by highlighting the unexpectedly high frequency of the H2 haplotype in Jordan, which diverges from patterns observed in European, Asian, and African populations. This distinction underscores the relevance of the regional genetic architecture in refining Alzheimer’s disease risk models.

### 1.1 Research Methodology

We conducted a comprehensive literature search across three major databases: PubMed (MEDLINE), Scopus, and Web of Science to identify relevant studies published between January 2000 and December 2024. Our search strategy incorporated several key terms, including “MAPT haplotype,” “Alzheimer's disease,” “tau,” “H1 H2,” “Jordan,” “population genetics,” “genetic risk modeling,” and “neurodegeneration.” We also searched for related terms such as “microtubule-associated protein tau,” “tau pathology,” “neurofibrillary tangles,” “pharmacogenetics,” “APOE,” “Middle Eastern populations,” and “haplotype frequency” using various combinations with Boolean operators.

We included peer-reviewed original research articles and reviews that reported MAPT haplotype frequencies in human populations, investigated associations between MAPT haplotypes and Alzheimer's disease, examined tau pathology concerning H1/H2 haplotypes, or focused on population-specific genetic variations. Only English-language publications were considered. We excluded conference abstracts, case reports, editorials, studies focusing solely on animal models without human relevance, articles not directly related to MAPT haplotypes or Alzheimer's disease, publications with insufficient methodological detail, and duplicate studies with overlapping datasets.

From the selected studies, we extracted relevant data including study population demographics, sample sizes, MAPT haplotype frequencies, geographic and ethnic distribution data, clinical and pathological outcomes, statistical associations with Alzheimer's disease risk, and interactions with other genetic factors, particularly APOE status. Our initial database searches identified numerous potentially relevant articles, which we screened first by title and abstract, then by full-text review. We also examined the reference lists of included studies to identify additional relevant publications.

We assessed the quality and relevance of included studies based on their study design, sample size, population characteristics, methodological rigor, and clarity of reported results. We prioritized studies with well-defined populations, adequate sample sizes, and robust genetic analysis methods. This review has limitations inherent to literature-based studies, as it may not capture all relevant research, particularly studies published in regional journals or non-English publications. As a narrative review synthesizing existing literature, we did not generate new experimental data or perform original statistical analyses.

## 2 MAPT GENE AND HAPLOTYPE BACKGROUND

H1 Haplotype: The H1 haplotype has been consistently associated with an increased risk of AD. The risk is greater in non-carriers of the APOE ε4 allele, indicating a tau-driven pathway that is independent of the amyloid β pathology-independent pathway in AD [11]. Several studies have reported that carriers of the H1 haplotype have an odds ratio of approximately 1.12 for AD risk, underlining a modest but significant association [2, 12]. Consequently, overexpression of MAPT in H1 carriers enhances tau build-up, which is central to AD progression, through tau phosphorylation and aggregation into neurofibrillary tangles, a core pathological feature of Alzheimer’s disease [2].

Epidemiological surveys have identified the H1 haplotype as a risk factor for AD [4]. This increased risk is particularly evident in subjects without the APOE ε4 allele, which is considered a major genetic risk factor for AD. These data would suggest that H1 acts *via* tau-associated pathways independent of amyloid beta pathology, highlighting a different mechanistic pathway in the pathogenesis of AD in this subset of patients [13]. Furthermore, the H1 haplotype affects the rate of cognitive decline in AD beyond the question of disease susceptibility. Longitudinal studies indicate that H1 carriers experience faster cognitive deterioration than non-carriers. This effect may reflect enhanced tau aggregation and neurofibrillary tangle formation, which are central features of AD [14]. Taken together, these observations support the role of the H1 haplotype in accelerating neurodegenerative processes and influencing disease progression.

The neurobiological mechanisms linking the H1 haplotype with AD progression involve the expression and function of the tau protein. For instance, the H1 haplotype is associated with increased expression of MAPT, including elevated levels of tau isoforms, in brain regions vulnerable to AD pathology. These isoform shifts facilitate tau aggregation in pathological structures, leading to neurofibrillary tangles, neuronal dysfunction, and eventual cell death. The influence of the H1 haplotype on tau an important route through which this genetic variant might promote disease, independent of amyloid β pathology [15].

The MAPT H1 haplotype is an important genetic factor in Alzheimer's disease because it modulates both disease susceptibility and cognitive decline. This haplotype contributes to AD pathology and may influence tau expression and aggregation pathways [2]. The association between the H1 haplotype and tau accumulation has prompted an investigation into whether it may affect the therapeutic response to tau-targeting agents, although no clinical evidence currently supports this application, and no direct therapeutic implications can be drawn from current observational data [2, 16].

H2 Haplotype: The H2 haplotype is considered protective with a lower risk of AD. Individuals with the H2 haplotype express MAPT at reduced levels and are thus associated with a reduced incidence of neurofibrillary tangle deposition and tau-related neurodegeneration [4]. H2 carriers have also demonstrated better performance in cognitive assessments than H1 carriers, supporting a potential role in slowing disease progression. H1 carriers further supported this haplotype in reducing the severity of disease progression. Several studies have described the H2 haplotype as protective and have suggested that it may modulate key mechanisms involved in tau pathology [17].

The H2 haplotype of MAPT is associated with a reduced risk of neurodegenerative diseases, particularly AD and Parkinson’s disease [18]. Studies have reported a lower risk of late-onset AD among H2 carriers, with a uniform odds ratio of approximately 0.90, indicating a protective association. This association is thought to result from lower MAPT expression levels in H2 carriers, leading to decreased tau accumulation and neurofibrillary tangle formation [19]. This risk reduction is believed to occur because of the lower levels of “tau protein” expression in carriers of H2, which lowers the deposition of neurofibrillary tangles in AD. Homozygous H2 individuals showed reduced total MAPT mRNA expression, which may contribute to lower susceptibility to tau-related neurodegeneration. Hence, they most likely protect against tau neurodegenerative devastating disability.

Carriers of the H2 haplotype performed better in cognitive assessments than those with the H1 haplotype, showing fewer neuropsychological deficits and overall better cognitive function, possibly reflecting a mitigating effect on neurodegeneration-related impairment. This pattern is supported by neuropathological findings showing that H2 carriers exhibit less severe tau pathology, including lower neurofibrillary tangle scores and other tau-related markers [20]. The protective effect of H2 extends beyond that of AD. In Parkinson’s, H2 has been associated with reduced disease risk, with an odds ratio of 0.76, suggesting a lower likelihood of cognitive decline and dementia in H2 carriers [2]. Similarly, outside AD, the H2 haplotype confers protection in PD, where the OR for reduced PD risk among carriers of H2 is 0.76, which in turn suggests a low probability of carriers developing cognitive decline and dementia due to PD [2, 19]. Overall, the protective association of the MAPT H2 haplotype across neurodegenerative diseases was consistently linked to reduced MAPT expression, lower tau burden, and more favorable cognitive outcomes. These findings reinforce the role of genetic variation in modulating neurodegenerative trajectories and may inform future approaches for identifying population-specific risk [21-23].

Although the H1 haplotype is generally associated with increased susceptibility to AD and other tauopathies, the H2 haplotype seems to be protective against these conditions. This differential association points to the fact that this contrast supports the view that haplotypes within the MAPT locus will be important for understanding their role in genetic predispositions to AD and may, in the future, facilitate further research into therapies targeted at the disease, which could inform future studies focused on risk stratification and disease mechanisms [24, 25].

## 3 POPULATION-LEVEL DISTRIBUTION OF MAPT HAPLOTYPES

MAPT haplotypes H1 and H2 modulate disease risk and progression through distinct mechanisms, particularly in their interaction with APOE status. The H1 haplotype is generally considered a major risk factor for AD, mainly because of its well-established relationship with higher MAPT expression, which in turn correlates with increased deposition of neurofibrillary tangles and heightened tau-related neurodegeneration [4]. It have been shown that H1 carriers run a higher risk of cognitive decline and earlier age of dementia onset than non-carriers or those carrying the H2 haplotype, indicating that H1 is a major factor in the disease trajectory between the MAPT H1 and APOE ε4 alleles and suggesting a compounding effect between H1 and APOE ε4 in shaping disease progression [2].

A major genetic risk factor for AD is the complex interaction between the underlying mechanisms of the disease, which involves the interaction of distinct molecular pathways contributing to neurodegeneration. Although APOE ε4 is highly related to amyloid-beta pathways, the presence of H1 points to another path driven by tau, which contributes to neurodegeneration independently of amyloid pathology [19, 26]. The tau-centric mechanism of the H1 haplotype indicates that even in the absence of the APOE ε4 allele, carriers of H1 are at a heightened risk due to tau dysregulation. Studies have shown that H1 carriers exhibit accelerated cognitive decline, worse tau pathology, and develop dementia within five years of Parkinson's disease diagnosis with an odds ratio of 12.1, highlighting the strength of the association between H1 and tau-driven neurodegeneration [26].

The H2 haplotype is common in Jordanian populations. It is linked to lower MAPT expression and reduced tau pathology. This pattern suggests that a genetic configuration may confer resilience against tau-driven neurodegeneration. This is important because it reduces the build-up of neurofibrillary tangles. It also helps to maintain the neuronal structure intact, especially in areas affected by Alzheimer’s disease. Our findings suggested a possible protective effect in certain populations. This aligns with the existing studies that connect H2 to slower cognitive decline and less severe clinical outcomes. These data are not conclusive but show a clear link. This supports the idea that the H2 haplotype acts as a genetic buffer in tau-related diseases [26, 27].

This attenuation of tau pathology suggests that the H2 haplotype could diminish cognitive impairment typical of AD, thereby minimizing neuropsychological deficits over time [28]. Effects of other genetic factors, such as APOE status, inflammation, and cancer. Other genetic factors, including the APOE status, influence parallel disease pathways that intersect with neurodegeneration. While the APOE ε4 allele drives amyloid-associated pathways, the presence of the H2 haplotype in this context may signal another protective mechanism that acts in concert with or independently of amyloid pathology operating alongside or independently of amyloid-related processes [26]. This finding indicates that there is an underlying genetic complexity in AD, given the possible ways through which diverse pathways could contribute to or prevent neurodegeneration in specific subsets of the population. These observations underscore the genetic complexity of AD and suggest that distinct mechanisms may confer risk or protection to different population subsets.

The distinct roles of the MAPT haplotype in AD have significant implications for personalized therapeutic stratification. For instance, in the absence of the APOE ε4 allele, elders with the H1 haplotype might benefit the most from certain interventions that target tau-related pathology without the complicating elements contributed by amyloid-beta pathways. Treatments structured to consider individual genetic backgrounds would thus offer a far more accurate way of addressing AD as knowledge about the various contributions of MAPT haplotypes increases. Stratifying treatment strategies based on MAPT haplotype status may improve therapeutic precision as the role of tau-related mechanisms becomes clearer [17].

In short, the H1 and H2 haplotypes of MAPT differentially confer the risk and progression of AD through interactions with genetic modifiers, such as APOE. This distinction enhances our understanding of the paths leading to the development of AD and opens future opportunities for the design of therapeutic modalities based on specific genetic constellations that promote personalized medicine in the management of ADs. This distinction improves the understanding of disease mechanisms and may support future efforts to develop genotype-informed therapeutic approaches for AD [4].

Research has shown that MAPT haplotypes might influence AD risk through complex mechanisms, influenced by other genetic modifiers, particularly the APOE status. The interaction of MAPT with APOE genes suggests that, in addition to an APOE-driven effect primarily influencing the amyloid beta pathways, a tau-driven MAPT-independent pathway may exist in AD. This further furthers the concept of individualized therapy, particularly for elderly individuals with the H1 haplotype, who do not have APOE ε4. These findings support the rationale for individualized therapeutic approaches, particularly in H1 carriers without the APOE ε4 allele, where tau-targeted strategies may be more appropriate [26, 29].

## 4 CROSS-POPULATION COMPARISON OF MAPT HAPLOTYPE FREQUENCIES

To place Jordanian findings in a broader context, we compared the H1 and H2 haplotype frequencies with those reported in neighboring Levantine populations and other global cohorts. As presented in Table **1** [30], the Jordanian population demonstrated a notably higher frequency of the protective H2 haplotype compared to European, East Asian, and African populations. A chi-square analysis comparing Jordan to a pooled set of non–Middle Eastern populations confirmed a statistically significant divergence in haplotype distribution (*p <* 0.0001), underscoring the genetic distinctiveness of this cohort and its relevance for future region-specific modeling of Alzheimer's disease susceptibility.

## 5 MECHANISTIC AND PROTECTIVE IMPLICATIONS OF THE H2 HAPLOTYPE

One of the relevant metrics that can be quantified to represent the genetic contribution of MAPT to AD is heritability, which is denoted as h^2^. The introduction of heritability estimates in the context of MAPT introduces a statistical framework in which specific variations in this gene can be attributed to AD risk [13]. This metric may be informative for the Jordanian population, in which the H2 haplotype is highly prevalent. Our review highlights a distinct haplotype distribution that may warrant future investigation of population-level genetic contributions to AD susceptibility. However, the potential of pharmacogenetic applications remains speculative in the absence of clinical or therapeutic data.

Genetic contributions to AD are increasingly being understood through the lens of heritability, especially relating to MAPT and its haplotypes (H1 and H2). Heritability (H^2^) [31]. In contrast, heritability (H^2^) allows the quantification of the proportion of phenotypic variance due to genetic variation. In this case, regarding the MAPT locus, heritability estimates clarified the extent to which genetic variation within a specific locus contributes to susceptibility to AD. This is especially significant in populations such as Jordanians, because the high prevalence of the H2 haplotype offers a great opportunity for studying genetic risk factors [32].

When the APOE gene region was not excluded, the heritability estimates dropped significantly. Although this points to the central role of APOE in AD risk development, the underlying contributions of other genetic factors, such as MAPT haplotypes, have also been recognized in disease susceptibility [2]. The involvement of MAPT in multiple neurodegenerative conditions supports its relevance in Alzheimer’s disease as well. Although the H1 haplotype is considered to confer an increased risk, the H2 haplotype is believed to play a protective role. The heritability study of these haplotypes further elucidates how they contribute to susceptibility to AD in a population-specific context. For example, the high frequency of the H2 haplotype among Jordanians indicates a peculiar genetic background that may modulate susceptibility to AD [33].

In any case, the heritability within Jordanian cohorts has great implications for AD risk. Individuals carrying the H2 haplotype present lower levels of MAPT expression and reduced neurofibrillary tangle formation, which are major pathological hallmarks of AD. These population genetic findings not only provide a framework to investigate regional disparities in AD risk but also offer a basis for exploring genotype-informed approaches. This may eventually contribute to the design of treatment strategies aligned with the genetic backgrounds of specific populations [16].

The reported MAPT haplotype distributions vary widely across populations. In European cohorts, the H1 haplotype is typically dominant, with H2 frequencies ranging between 15% and 25% [4]. In East Asian populations, H2 is largely absent, whereas in African populations, its distribution is more variable. The apparent overrepresentation of the H2 haplotype in the Jordanian population, reported in some studies to exceed 40%, deviates from these global patterns [34-36]. Although further large-scale genotyping is needed to confirm this trend, these observations suggest potential regional differences in genetic architecture that may be relevant for disease susceptibility modeling and therapeutic planning. Including such comparisons strengthens the interpretive value of the observed distribution and places Jordanian data in a clearer context [37-39].

The interaction between the haplotype status of MAPT and personalized strategies for therapy in AD management. For example, in elderly subjects carrying the H1 haplotype and not carriers of the APOE ε4 allele, therapies targeting tau-related pathways may be particularly beneficial [40]. By contrast, individuals with the protective H2 haplotype may require less aggressive therapeutic approaches, reflecting a lower genetic risk profile. This highlights the importance of integrating genetic information into personalized care models to maximize treatment outcomes [3].

Heritability estimates associated with MAPT provide insight into the genetic underpinnings of Alzheimer's disease. These two haplotypes, H1 and H2, contribute to the genetics of AD, highlight the population-specific modifiers of the disease, and may inform future research into targeted intervention strategies. Further studies on heritability in cohorts, such as Jordanians, will help elucidate regional variations in AD support efforts toward developing genetically informed therapeutic approaches [2].

## 6 RELEVANCE TO RISK MODELING AND STRATIFIED RESEARCH DESIGN

Recent studies have further refined this understanding by identifying sub-haplotypes within H1 and H2 that further influence AD risk and tau expression. While some H1 sub-haplotypes are associated with particularly high MAPT expression, leading to pronounced tau accumulation, certain H2 sub-haplotypes appear to confer greater resistance to tau pathology [41].

Recent findings in the study of haplotype structures of the MAPT gene revealed that H1 and H2 sub-haplotypes exist and provide new insights into their roles, both in AD risk and tau expression, as shown in Table **2**. A comparative summary of the selected MAPT sub-haplotypes, their expression patterns, tau pathology profiles, cognitive effects, and interaction with APOE ε4 is presented in Table **1**. These findings contribute to a more detailed understanding of the genetic architecture of neurodegenerative diseases and support the need for sub-haplotype level analyses in future research [4, 17].

Recent reports have revealed that both H1 and H2 contain sub-haplotypes that confer variable risks of AD and other tauopathies. Some H1 sub-haplotypes seem to be associated with increased MAPT expression, which in turn correlates with higher tau brain burden, potentially contributing to an increased risk of tau-driven neurodegeneration [4]. In contrast, some H2 subhaplotypes appear to protect against tauopathy through the maintenance of lower levels of tau expression or favorable ratios of tau isoforms. Such variations illustrate the genetic complexity through which genetic factors modulate tau dynamics, and how they may influence AD progression [15, 42].

Furthermore, certain sub-haplotypes under the H1 haplotype have been associated with a marked increase in tau protein expression, thereby enhancing tau pathology. Protective H2 sub-haplotypes might reduce tau accumulation due to mechanisms involving either diminished expression or alterations in tau aggregation properties [43]. These observations underscore the importance of sub-haplotype-level variations in shaping the disease mechanisms. Other genetic modifiers, such as APOE, interact with MAPT sub-haplotype effects to further increase complexity. Although APOE mainly affects amyloid-β pathways, the impact of MAPT seems to drive tau-related pathologies independent of APOE status [43].

This suggests that individuals with certain sub-haplotypes in the MAPT may require therapeutic strategies that address both amyloid- and tau-related pathways [44]. These sub-haplotypes invite detailed investigations into their contributions to the risk of AD and tau pathology. Elucidation of the interaction between genetic variants, environmental factors, and other genetic modifiers is crucial for developing personalized medical strategies to prevent or alleviate AD [45]. For example, therapies targeting tau dynamics by reducing tau levels, altering aggregation behavior, or supporting neuroprotective responses [46, 47].

In summary, the identification of these sub-haplotypes on the MAPT locus opens perspectives for the understanding and investigation of Alzheimer's disease risk and tau pathology. The subtle genetic architecture evident in these the relevance and potential of incorporating the genetic makeup of an individual in the assessment of disease risk and therapeutic development. Continued investigation of MAPT sub-haplotypes is necessary to define their role in neurodegenerative diseases and may support the future development of targeted, genotype-informed therapeutic strategies [48].

## 7 THERAPEUTIC ASPECT OF RESEARCH ON THE MAPT HAPLOTYPE

The consequences of MAPT genetics stretch beyond the bounds of risk association and extend to informing potential therapeutic strategies. Treatment approaches that target tau expression modulated by functional MAPT haplotypes could be developed by directly targeting genetic profiles, such as tau-lowering therapies for H1 carriers or drug regimens based on interacting APOE and MAPT haplotype effects. Targeted interventions using a precision medicine approach in AD care might reduce tau-mediated neurodegeneration in populations at risk, including carriers of specific H1 sub-haplotypes with a very high risk [17]. A mechanistic overview of MAPT haplotypes, tau-related molecular effects, therapeutic implications, and APOE interactions is presented in Table **3**.

The therapeutic implications of the investigation of MAPT haplotypes transcend simple risk assessments, especially in the context of AD, and involve targeted strategies for treatment. The role of MAPT haplotypes in modulating neurodegeneration is important for understanding genetic influences on tau expression. These findings support the development of therapies tailored to MAPT genetic profiles, contributing to the broader goal of precision medicine for disease management [17, 49].

The tau gene, MAPT, encodes the tau protein and is widely regarded as a central player in neurodegenerative diseases, including AD. The H1 haplotype, which is associated with increased tau expression, is associated with a higher risk of neurodegeneration. Modulation of tau expression through therapeutic intervention among carriers of the H1 haplotype offers a new avenue of opportunity for therapeutic benefits against tau-mediated pathologies [50]. Potential strategies include immunotherapies targeted at enhancing immune responses against pathological tau species, and kinase inhibitors, including those directed against GSK-3β, to reduce tau hyperphosphorylation and aggregation. Such treatments may be particularly beneficial in carriers of high-risk H1 sub-haplotypes, in whom the risk of tau-related pathology is marked [51, 52].

The interaction between MAPT haplotypes and APOE genotypes suggests that there is a need for tailored therapeutic strategies based on haplotype/genotype backgrounds. For instance, carriers of the H1 haplotype that do not have the APOE ε4 allele may respond differently to treatment than those with both genetic risk factors [2]. This further reinforces the idea of precision medicine approaches that consider the genetic backgrounds of both MAPT and APOE when designing treatment regimens. Such regimens might improve therapeutic efficacy through actions on both amyloid- and tau-related pathways [3, 53].

Precision medicine involves the provision of personalized treatment to patients based on their genetic fingerprints. Characterization of specific MAPT sub-haplotypes, along with determination of the associated risks, will help clinicians build particular interventions based on understanding the neurodegenerative mechanisms at play. For example, carriers of high-risk H1 sub-haplotypes might be indicated as first in line for tau-directed therapies. In contrast, individuals with protective H2 sub-haplotypes might be best served by monitoring rather than aggressive treatment [54]. Such genetic profiles could also yield pharmacogenetic information that may be useful in the selection and dosing of drugs, thus optimizing therapeutic responses and minimizing toxicity [40].

Ongoing clinical trials testing tau-targeted therapies, including monoclonal antibodies and small molecules, are expected to provide important insights into their efficacy depending on the genetic background. The results from such studies will help refine the treatment protocols based on MAPT haplotypes and ensure that the treatments are tailored to the patient's genetic predisposition to AD. Further in-depth research into the interaction between MAPT haplotypes, APOE status, and other genetic modifiers will lead to a holistic understanding of AD pathogenesis [47, 55].

This is the only way this integrated approach can target tau pathology in concert with other contributing factors, including the build-up of Aβ. In general, novel investigations into the haplotypes of MAPT provide great perspectives for the treatment of neurodegenerative diseases tailored to a person's genetic background. Thus, by being in a position to modify tau expression and considering specific genetic risks, researchers and scholars will be in a position to rectify the effectiveness of remedies for vulnerable individuals with AD [56]. Future studies focusing on haplotypes of the MAPT gene and their interactions with other genetic variants will be critical for the development of precision medicine and for enhancing therapeutic responses across heterogeneous patient groups [4, 24].

Investigation of MAPT haplotypes, especially H1 and H2, has thus identified their importance in the risk, progression, and therapeutic potential for AD. Genetic risk represented by the H1 haplotype has been sufficiently established, mainly through its association with elevated MAPT expression, tau aggregation, and cognitive decline. In contrast, the H2 haplotype exerts protection through its dampening action on MAPT, thereby acting as a protective modifier against tau-driven neurodegeneration. These haplotypes influence disease susceptibility and exert therapeutic effects.

Refinements of genetic research in sub-haplotypes within H1 and H2 further illuminate the pathogenesis of AD and provide novel targets for precision medicine. The interaction between haplotypes of the MAPT gene and other genetic modifiers, such as APOE, sharply focuses on the complexity of the genetic architecture in AD and supports individualized therapeutic planning. Genetic profiles for precision medicine can enhance therapeutic outcomes by offering targeted interventions that address both the tau- and amyloid-related pathways.

Tau-targeted therapies include immunotherapies, kinase inhibitors, and monoclonal antibodies. This represents one light in the tunnel for arresting neurodegeneration in high-risk populations, especially H1 carriers. The protective influence of H2 sub-haplotypes provides insight into mechanisms that may inspire novel neuroprotective strategies. Although no direct clinical or pharmacogenetic outcomes were assessed in this study, the distinct haplotype distribution observed may have implications for future therapeutic stratification in populations with similar genetic profiles [57]. Further investigation is needed to determine whether such distribution patterns correspond to differential treatment responses or disease trajectories [58].

Given that the Jordanian cohort has a high prevalence of the H2 haplotype, the heritability of the MAPT study has wide-ranging implications for regional pharmacogenetics and public health strategies. Genetic contribution of MAPT haplotypes in distinct populations. These population-level genetic observations may inform future research aimed at refining regional risk models; however, therapeutic applications require clinical validation.

In general, haplotypes within MAPT, including H1 and H2, provide a platform upon which further genetic underpinning of Alzheimer's disease and other tauopathies may proceed [4]. Additional research on genetic-environmental-molecular interactions influencing MAPT and tau dynamic expression continues to be of critical importance for refining therapeutic approaches. Integration with precision medicine may support the translation of these findings into improved patient care, with more targeted interventions and optimized outcomes in diverse populations [59]. Endogenous stress response systems, such as the Nrf2-regulated network, help cells resist oxidative injury. Vita genes are a group of genes controlled by Nrf2. They help maintain the redox balance and support mitochondrial health. These systems can interact with genetic factors [60]. For example, MAPT haplotypes influence vulnerability to tauopathy. The H2 haplotype may help to lower tau expression. It can work with Nrf2 mechanisms to reduce oxidative stress and protect neurons [61, 62]. This model supports the findings of previous studies. It highlights how crucial stress adaptation pathways are in neurodegeneration.

The markedly high frequency of the H2 haplotype observed in this Jordanian cohort raises the possibility of region-specific modulation of AD risk that has not been captured in broader genomic datasets [63]. Given the known association between H2 and reduced tau pathology, this pattern may reflect a distinct genetic buffer in this population. Such findings underscore the importance of incorporating underrepresented populations into genomic studies of neurodegeneration [50]. They also support the need for regional stratification in the design and interpretation of therapeutic trials targeting tau-mediated pathology. These insights contribute to closing existing gaps in the population-genetic landscape of Alzheimer’s disease and inform efforts to expand genetic diversity in AD research [47].

However, we recognize that no functional, biomarker, or clinical data were included in this review to establish a direct link between haplotype distribution and AD-related outcomes such as tau pathology or cognitive performance. The associations discussed are drawn from published sources and are not supported by imaging or longitudinal data in the Jordanian population. As such, our conclusions are interpretive and highlight the need for future studies that integrate genetic profiling with clinical and neuropathological endpoints.

This review is based on existing literature and does not include original clinical, biomarker, or genomic data. As such, interpretations are limited to associations reported in prior studies, and no causal conclusions can be drawn regarding MAPT haplotypes and Alzheimer’s disease outcomes. Additionally, while the emphasis on the Jordanian population addresses a recognized gap in genetic representation, comparative data remain limited. They may not capture the full variability across the broader Middle Eastern region. The absence of functional validation and the reliance on reported haplotype frequencies also limit generalizability. Future work should integrate genotyping with imaging, biomarker, and clinical outcome measures to determine the translational relevance of MAPT variation in diverse populations.

This review is narrative in nature and does not include original genetic, imaging, or biomarker data. All MAPT haplotype associations discussed are drawn from published literature and not from direct experimental or clinical observations. While we highlight the potential significance of the H2 haplotype in regional cohorts such as Jordan, these interpretations remain speculative in the absence of functional validation. No PET imaging, cerebrospinal fluid analysis, or cognitive testing was conducted in this population to link haplotype status with Alzheimer’s disease outcomes directly. Future studies are warranted to integrate genotypic information with clinical, neuroimaging, and biomarker data to assess the translational relevance of MAPT haplotype variation in Middle Eastern populations.

## 8 STUDY LIMITATIONS

This review is interpretive and does not include original genotyping, biomarker, imaging, or clinical data. All MAPT haplotype associations discussed are drawn from published literature. While the overrepresentation of the H2 haplotype in the Jordanian population presents a potentially important pattern, no functional validation was conducted. The findings are hypothesis-generating and should not be used to guide therapeutic decisions without further study.

## CONCLUSION

Variation in the MAPT gene remains a key focus in understanding genetic susceptibility to Alzheimer’s disease. The contrast between the H1 and H2 haplotypes—particularly their association with tau burden—has been consistently reported across populations. This article draws attention to the underrepresented genetic data from the Middle East, with a specific focus on the observed overrepresentation of the H2 haplotype in Jordan. While the protective potential of this haplotype has been described in other cohorts, its prevalence in this regional context may carry implications for future study designs that account for geographic and ethnic variability in genetic risk.

This work does not present original functional or clinical data and does not draw therapeutic conclusions. Instead, it serves to consolidate the available evidence and highlight areas where further investigation is warranted. Future studies combining genotyping with clinical, imaging, and biomarker data will be essential to determine whether MAPT haplotypes can inform stratified models of disease risk or intervention.

Understanding the regional distribution of MAPT haplotypes is a step toward addressing current gaps in representation in Alzheimer’s genetics. Broader inclusion of diverse populations is needed to improve the accuracy and equity of disease modeling and treatment development. All genetic, clinical, and population-level data referenced in this review are drawn from published sources and not derived from original research conducted by the authors. This article draws attention to the underrepresented genetic data from the Middle East, with a specific focus on the observed overrepresentation of the H2 haplotype in Jordan. While the protective potential of this haplotype has been described in other cohorts, its prevalence in this regional context may carry implications for future study designs that account for geographic and ethnic variability in genetic risk. These population-level genetic observations may inform future research aimed at refining regional risk models, but therapeutic applications require clinical validation. As large-scale consortia expand to include underrepresented populations, Middle Eastern cohorts like Jordan may prove pivotal in refining global genetic risk maps for Alzheimer’s disease.

## Figures and Tables

**Table 1:** Global MAPT haplotype frequencies across regional populations.

**Population**	**H1 (%)**	**H2 (%)**	**Sample Size**
Jordan	61.5%	38.5%	312
Lebanon	65%	35%	200
Palestine	63%	37%	180
Europe	75%	25%	500
East Asia	98%	2%	450
Africa	70%	30%	300

**Note**: H1 and H2 haplotype distributions are presented as percentages across Jordan, neighboring Levantine populations (Lebanon and Palestine), and representative global groups. A chi-square comparison between the Jordanian population and the pooled non–Middle Eastern populations (Europe, East Asia, and Africa) showed significant differences in distribution (*p <* 0.0001) [9, 30].

**Table 2:** Summary of functional characteristics, tau pathology profiles, cognitive outcomes, APOE ε4 interactions, and population prevalence associated with selected MAPT sub-haplotypes.

**Sub-Haplotype**	**Associated MAPT** **Expression**	**Tau Pathology (↑/↓)**	**Cognitive Impact**	**APOE ε4** **Interaction**	**Known Population** **Frequency**
H1c	↑ High	↑ Strong aggregation	↓ Accelerated decline	Yes	Common in Europeans
H1e	↑ Moderate	↑ Mild aggregation	Neutral	No	Reported in mixed ancestry
H2a	↓ Low	↓ Reduced tangles	↑ Preserved cognition	No	High in the Jordanian cohort
H2b	↓ Very Low	↓ Minimal pathology	↑ Stable cognition	No	Rare globally

**Note**: Sub-haplotypes within the H1 and H2 lineages exhibited distinct MAPT expression patterns, influencing tau protein aggregation and downstream cognitive effects. H1c, for example, is associated with elevated MAPT expression and accelerated neurofibrillary tangle formation, particularly in APOE ε4 non-carriers, consistent with studies linking it to early cognitive decline [12, 16, 29]. In contrast, H2a and H2b sub-haplotypes show reduced MAPT expression and less tau pathology, with stronger cognitive preservation, particularly in populations such as Jordanians, where H2 prevalence is higher [21, 34, 35]. These functional differences underscore the genetic complexity of Alzheimer’s disease and support the rationale for sub-haplotype-informed precision medicine.

**Table 3:** Summary of the functional consequences of MAPT haplotypes (H1 and H2), their interaction with key molecular pathways, and implications for therapeutic intervention.

**MAPT Haplotype**	**Tau Pathology** **Signature**	**Affected Molecular** **Pathways**	**Suggested Therapeutic Targets**	**APOE ε4 Interaction**	**Translational** **Relevance**
H1	Hyperphosphorylation, aggregation	↑ GSK-3β, ↓ Autophagy, ↑ Microglial activation	GSK-3β inhibitors, tau immunotherapy	Synergistic tau/ amyloid pathology	High-risk AD progression profile
H2	Reduced tau burden	↓ MAPT expression, ↑ Isoform balance	None required or neuroprotective support	Independent of APOE status	Low-risk, potentially protective
H1/H1 (homozygous)	Accelerated NFT formation	↑ MAPT mRNA, ↑ Exon 10 inclusion	Splice-modulating antisense therapy	Enhanced vulnerability	Target for early tau-directed intervention
H2/H2 (homozygous)	Minimal pathology	↓ Tau expression, ↓ Neuroinflammation	Biomarker monitoring only	No impact	Genetic buffer in precision stratification

## LIST OF ABBREVIATIONS

AD: Alzheimer’s Disease
APC: Article Processing Charge
APOE: Apolipoprotein E
CSF: Cerebrospinal Fluid
DNA: Deoxyribonucleic Acid
GSK-3β: Glycogen Synthase Kinase-3 Beta
H1: MAPT Haplotype 1
H1c: MAPT Sub-haplotype H1c
H1e: MAPT Sub-haplotype H1e
h^2^: Heritability Estimate
H2: MAPT Haplotype 2
H2a: MAPT Sub-haplotype H2a
H2b: MAPT Sub-haplotype H2b
MAPT: Microtubule-associated Protein Tau
mRNA: Messenger RNA
NFT: Neurofibrillary Tangle
Nrf2: Nuclear Factor Erythroid 2-related Factor 2
OR: Odds Ratio
PD: Parkinson’s Disease
PET: Positron Emission Tomography
RNA: Ribonucleic Acid
Scopus: Scopus Database
Tau: Tau Protein
WoS: Web of Science
